# Role of Snowpack-Hydrometeorological Sensors for Hydrogeological System Comprehension inside an Alpine Closed-Basin

**DOI:** 10.3390/s22197130

**Published:** 2022-09-20

**Authors:** Michele Mondani, Martina Gizzi, Glenda Taddia

**Affiliations:** Department of Environment, Land and Infrastructure Engineering (DIATI), Politecnico di Torino, 10129 Torino, Italy

**Keywords:** hydrogeology, climate change, groundwater monitoring, mountain spring, Italy

## Abstract

Groundwater resource assessment and forecasting in mountain areas requires the monitoring of two conditions, local meteorological conditions, and springs’ groundwater parameters. The reliability of the monitoring data and conditions are linked to the technical instrumentation, multiparametric probes, and sensors. This paper presents a set of attractive tools and sensors for springs’ groundwater resource monitoring and assessment in mountain basins. Data from the combination of weather station sensors with spring flow-rate instruments, installed in the alpine Mascognaz basin, can guarantee an entire understanding of how one set of parameters can affect other results, defining consequential cause-and-effect relationships. Since a large part of the Alpine groundwater bodies are exploited for drinking purposes, understanding the evolution of their rechange processes requires making the right economic and instrumental investments aimed at using them according to forecast predictions and sustainable development goals.

## 1. Introduction

Groundwaters represent the most abundant source of fresh water on Earth, accounting for approximately 97% of non-frozen water. It is an important natural resource that has greatly contributed to social development [1]. Population growth, economic development, and consumption patterns are responsible for the increase in groundwater exploitation trends: global water demand has increased by 600% over the past 100 years, corresponding to an annual increment rate of 1.8% [2]. Groundwater resources have become increasingly vulnerable due to phenomena such as urbanization, agricultural exploitation, industrial production, and intensive farming processes [3].

The alpine climate system, as well as the hydrogeological recharge mechanisms, are characterized by a network of complex interactions. The negative impacts on such recharge processes deriving from climate change are still largely unknown. Therefore, examining how groundwater storage mechanisms have changed in response to both climate-driven and anthropogenic effects is becoming increasingly crucial. In doing so, long-term systematic measurements of local meteorological conditions and springs’ groundwater parameters, such as spring discharge, temperature, and electric conductivity, can be used to investigate important features of the aquifer and provide essential data needed to evaluate changes in the groundwater resource over time, to develop models, and forecast trends, eventually designing and monitoring the effectiveness of management and protection programs [4,5,6].

Several authors have started to analyze the influence of climate change on water resource availability, with particular regard to groundwater storage mechanisms [7,8]. In different areas of the world, aquifer depletion phenomena have been detected both on a regional and local scale [9,10]. These turn out to be mostly determined by a recent evolution of the pluviometric characteristics, which appear to be defined by shorter and more intense events with a lower frequency which results in fewer rainy days per year [11,12,13]. Furthermore, it decrease in solid precipitation accompanied by a temporal shift as regards the melting of the snow has been described, leading to a premature and damped spring discharge response in mountain basins. [14,15,16,17,18] studied the importance of the snow melting phenomenon for the aquifer recharging phase in mountain environments. The increase in air temperature and solar radiation are driving factors for the remobilization of water resources from glacier and permafrost sectors, which sometimes compensates for the reduction of water from solid and liquid precipitations [19,20].

According to [21,22,23], the combination of weather station sensors with spring flow-rate instruments allows for properly correlation of the input and output data values of the hydro-meteorological system’s variables, providing information about spring recharge mechanisms and their recent evolution. As reported above, long-term systematic measurements are essential for comprehending the hydro-meteorological system evolution of a specific mountain basin, by being able to analyze the depicted trends from a statistical point of view [24,25]. Furthermore, the data temporal resolution is also important to observe the seasonality of the parameters and, even more precisely, the hourly and daily behavior of the monitored atmospheric phenomena [26,27,28]. Increasingly sophisticated solutions are then needed to improve the quality of the data obtained [29,30].

Considering the described context, this paper aims to define the potential of using data from a combination of multiparametric probes and sensors for meteorological conditions monitoring and groundwater resource assessment in an alpine mountain area, such as the Mascognaz basin (Aosta Valley, Northwestern Italy). In the literature, several cases of spring monitoring coupled with weather monitoring are described [31,32,33,34]. However, the distance between the hydrogeological monitoring site (spring) and the meteorological stations in place is so great that the results from the subsequent analyses are not reliable.

In the Mascognaz station, the snow and meteorologic sensors are clustered within a unit station named the Snowpack-Weather Station (SWS), which is 700 m uphill with respect to the two springs’ hydrometric stations. The step of instruments and sensors implemented can bring to light data that reflect the recent evolution of hydrogeological trends, combined with meteorological ones. The presented instruments**’** equipment is of primary importance. First, for the characterization of the rainfall regime as a function of climatic conditions. Second, the springs, which reflects groundwater resource availability, are strongly correlated to precipitation amounts and cryosphere melting processes. The setup of the Mascognaz weather station, alongside common atmospheric monitoring sensors, includes the monitoring set for the snowpack analysis. This allows a more in-depth analysis of the hydrogeological balance, as a large water contribution could derive from the melting of the snow mantle [35,36]. Besides, the potential of the proposed Snowpack-Weather Station (SWS) will be shown by describing the hydrometric set-up applied to the case study to close the hydro-meteorological circuit. This also allows observation of the correlations between the spring behavior of the precipitation and melting phenomena of the snowpack [37], and understanding of the systematic link between the hydrogeological inputs and outputs. Therefore, underlining the importance of the monitoring and data collection activities through various instruments with the aim of studying the environmental evolution of hydrometeorological systems represents one of the aims of the work. Data and results produced by the different monitoring technologies turn out to be useful to mountain communities that are interested in understanding groundwater characteristics according to climatic and meteorological variations for sustainable water resources exploitation and management.

## 2. Materials and Methods

### 2.1. The Mascognaz Spring System Monitoring

The SWS and springs stations had to be correctly located inside a sufficiently closed hydrogeological basin, in which the hydrogeologic inputs being monitored are representative of hydrogeologic processes occurring within the studied area. 

**Figure 1:**
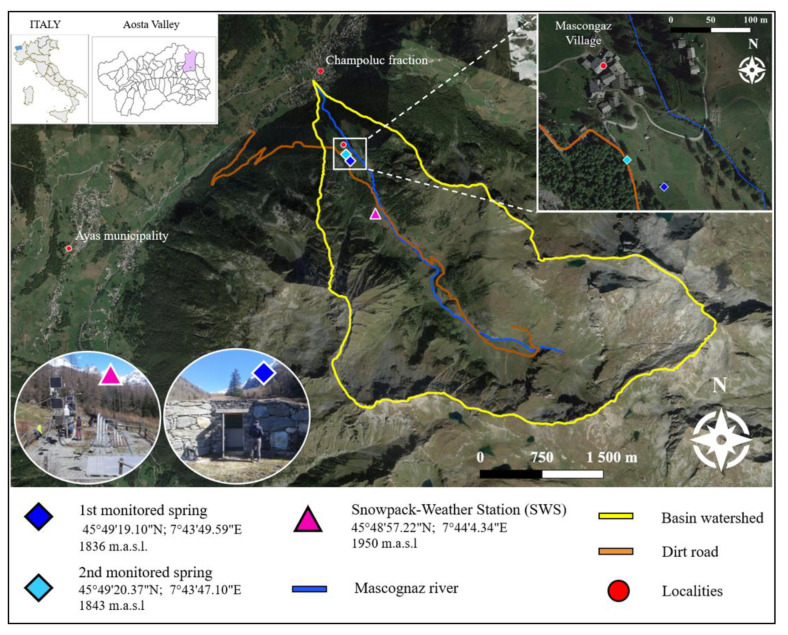
Mascognaz 1 and 2 springs and experimental meteorological station locations.

The site chosen to locate the experimental equipment is Mascognaz, which is in the Ayas municipality, near Champoluc, (Aosta Valley), as presented in Figure 1. The Mascognaz river basin ascends in the South-East direction; its extension is around 10 km^2^. The valley floor is partially covered by quaternary deposits, which store the porous aquifer. This is exploited for drinking purposes by means of an aqueduct system that brings water to Champoluc municipality.

The Mascognaz experimental Snowpack-Weather Station position is 800 m upstream of the Mascognaz village, on the left side of the Mascognaz river.

The SWS station location is easy to reach through a dirt road that connects the regional Aosta Valley road n °45 of the Mascognaz village. The same dirt road continues along the valley reaching few thalweg cottages (Figure 1). During winter the site is easily accessible by snowmobile or snowshoes; it is not placed within an avalanche risk area, and it presents snow coverage representative enough for the studied area. 

The area is also well monitored by other meteorological stations and manual surveys of the snowpack, which are useful in order to have a wide network of information to be compared to the values recorded by this case study setup.

The Mascognaz 1 and 2 springs were equipped with new instruments and multiparametric probes on 13 October 2010. While the Mascognaz experimental meteorological (SWS) station is placed a few hundred meters above the village, the two springs are located within the valley’s urbanized area, precisely, they are on the upper side of this area (Figure 1). They are located on the hydrographic left, respectively at 27 m and 10 m above the main stream, both about 80 m away from it. Due to their positions, they only partially collect the surface waters drained by the Mascognaz river. Both springs are monitored by employing a level, temperature, and electrical conductivity sensor.

Ayas municipal staff provide full collaboration for spring and weather monitoring. During normal routine checks, they guarantee the proper functioning of the springs’ probes and station devices.

### 2.2. Snowpack-Weather Station (SWS) Setup

The SWS station is composed of two clusters of sensors, some of which are attached to two anti-lighting steel towers and others are installed separately inside the SWS ranch perimeter. The first cluster, regarding the general atmospheric conditions, is installed by the Italian company Corr-Tek Idrometria S.r.l., San Giovanni Lupatoto, Italy, and the other one, regarding the snowpack conditions, is installed by an Austrian company, Sommer GmbH & Co. KG., Hörstel, Germany.

**Figure 2:**
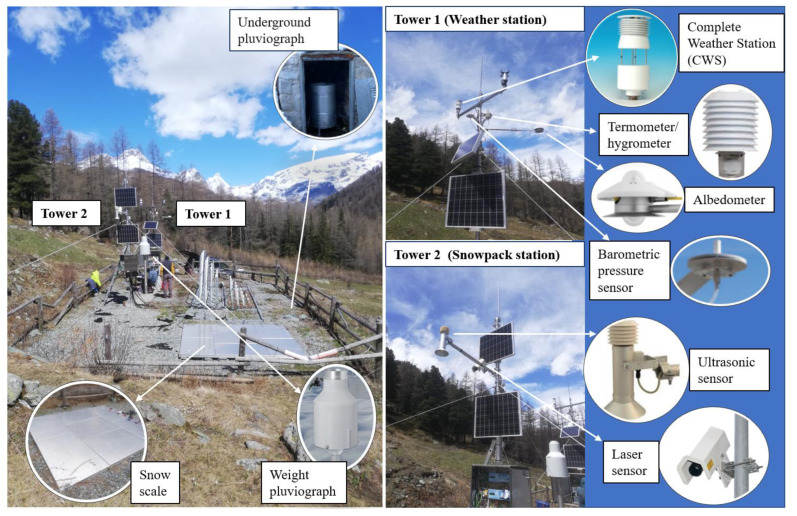
SWS arrangement and equipment.

Thus, Corr-Tek Idrometria S.r.l., San Giovanni Lupatoto, Italy sensors monitor weather parameters by means of Tower 1, underground pluviograph, and weight pluviograph, while Sommer GmbH & Co. KG., Hörstel, Germany, instruments monitor snowpack evolution through Tower 2 and snow scale (Figure 2).

Snowpack-Weather Station, via GPRS (General Packet Radio Service) connection, sends the information relating to the monitored parameters in 10-min time steps to the Functional Center. Sensors record multiple measurements during the 10 min and send the average value calculated on the individual values. In detail, the Corr-Tek Idrometria S.r.l., San Giovanni Lupatoto, Italy, is equipped with two SIMs while that of the Sommer GmbH & Co. KG., Hörstel, Germany, is equipped with a single SIM in GPRS.

The recorded data can be monitored in real-time from the indicator site by the two installers, who use this system to check that all the instruments are working correctly. Otherwise, they are the first to notice and warn users to identify what the problem is in the field before intervening with the operators. The data are visualized and controlled by means of the OTT Hydras 3 software package, provided by Corr-Tek Idrometria S.r.l., San Giovanni Lupatoto, Italy.

#### 2.2.1. Corr-Tek Meteorological Sensors

In this paragraph, meteorological equipment is described and the main features for each device are presented in Table 1.

Temperature and relative humidity (RH) are both recorded by the hygrometer. It is composed of two sensors within a cylindrical probe inserted inside a box that allows air ventilation. These two parameters are also detected by the CWS (Complete Weather Station), which encloses a precision triple-element Thermistor that reveals temperature values with high accuracy and stability. RH sensor of CWS is a capacitive polymer sensor, very resistant to wetting, dust, dirt, and environmental chemicals. CWS also detects wind speed and wind direction using a sonic anemometer, which operates according to the principle that the speed of the wind affects the time it takes for sound to travel between the pairs of transducers. Besides, CWS Internal compass allows correction of the wind direction according to the variation of magnetic North.

CMA 6 albedometer is an ISO First Class albedometer that uses two CMP 6 pyranometer detector assemblies built into a single housing. An integrated glare screen prevents direct illumination of the lower domes at sunrise and sunset and a screw-in drying cartridge keeps the interior free from humidity. A mounting rod is fitted to provide easy attachment to a mast. 

The Model 61,302 Barometric Pressure Sensor offers extremely high accuracy over a broad range of pressures and temperatures and since it is mounted outdoors, it has been protected with a discoidal weatherproof enclosure. Barometric pressure values can be cross-checked by means of CWS, which is able as well to provide measures of this parameter by exploiting a piezoresistive pressure sensor. 

The OTT rain gauge operates with a weight-based operating principle. A load cell reads the weight increase and an algorithm transforms it into mm of precipitation. The instrument has very high accuracy in both light and heavy rainfall. It is also able to measure solid precipitations without the need of a heating element, therefore, it can be exploited also in the case of remote installations.

The pluviograph DQA030 is a sensor for measuring the amount of rain. The measuring device is made up of a collecting cone and a double tank toggle connected to a magnet that activates a reed relay (option with two reed relays) which generates a pulse that can be counted from an external counter; each pulse corresponds to 0.2 mm of rain (options from 0.1 to 0.5 mm). This pluviograph is located underground according to the experimental purpose of recording the exact moment of snow melting and release, which is very useful to assess aquifer recharge beginning and to quantify the rate of water released from the snowpack. 

All the devices listed above are connected to the OTT netDL1000 multifunction data logger, which is installed inside the OTT MetSystem lockable control cabinet placed in tower 1. OTT netDL 1000 is produced by OTT Hydromet GmbH for data reading. It represents the main element of the data acquisition station. It is a versatile unit that can be used for applications in the hydrometry field, but also for generic data acquisition and transmission applications, for example in sectors such as meteorology and agrometeorology [23]. Its size is quite compact (23.2 cm × 12.4 cm × 8.5 cm), and it can operate within a wide range of temperatures (−40 °C to 70 °C), making this tool very suitable for a high mountain environment. This data logger is equipped with standardized ports communicating with sensors by several interfaces (SDI-12, RS-485, Modbus RTU, analog-in, impulse input, status input) and it owns different transmission protocols (HTTPS, HTTP, SMTP, FTP) and data formats, including XML. Parallel processing of the data from all connected sensors makes short sampling intervals possible. Moreover, the integrated web server allows access to the datalogger using standard browsers. A SIM is provided to the device to transmit data to the server by means of three available mobile internet network systems: GSM, GPRS, and 3G.

#### 2.2.2. Sommer GmbH & Co. KG Snowpack Sensors

The operating principle of the Snow Scale is based on the measurement of the load cells. The sensor consists of seven perforated surfaces (2.8 m × 2.4 m) and each surface has dimensions of 0.8 m × 1.4 m. The central panel and the six panels surrounding allow water to seep through the sensor. The percolation of the water minimizes the thermal differences between the sensor and the panels that surround it to reduce interference on the central panel, where the snow water equivalent (SWE) is measured. This system allows an accurate measurement even during periods of heavy snowfall with consequent abundant accumulations. The overall uncertainty of the Snow Scale is expressed with percentage (Table 2), since the standard deviations of measurements grow according to the amount of snow on the scale.

The combination with a snow-depth sensor (USH-8 for example) allows the average density of the snow to be calculated simply and easily. The pressure-compensation capillary integrated into the sensor cable reliably smooths out fluctuations in barometric pressure, thus preventing measurement errors. The Snow Scale is an important instrument for determining the water content of a layer of snow. This information is of great significance to water-supply utilities and hydrologists in the forecasting of high-water levels.

The measuring principle of the Ultrasonic Sensor (USH-8) is based on determining the transit time of an ultrasonic pulse. The sensor transmits many impulses to the snowpack and subsequently receives the reflected signals. Based on the required transit time of the ultrasonic signals, the sensor calculates snow depth. The processing time of the ultrasonic pulses is greatly influenced by the air temperature. For this reason, the snow depth sensor also has an integrated temperature measurement sensor. This takes into account the influence of the air temperature on the processing time of the ultrasonic signals when calculating the snow depth. The calculation takes place directly by the sensor setting, so that the output signal provides the correct snow depth measure. In this way, more reliable measurement results are obtained, and accuracy increases to 0.1% on the measured value (Table 2).

The laser sensor (SHM 30) detects the snow depth by analyzing the intensity of waves (signal strength), the number of waves, and the vector to obtain precise measurements. The precise distance is determined by using five different frequencies. The repetition of measurements provides higher accuracy on difficult targets, or during precipitation events. For snow depth measurements, the longest time interval is selected in order to measure filtering out noises.

All snow sensors are connected to tower 1 by means of reinforced cables permitting the data transmission to OTT netDL 1000 datalogger and the power supply from the electric control unit.

### 2.3. Hydrometric Equipment

**Figure 3:**
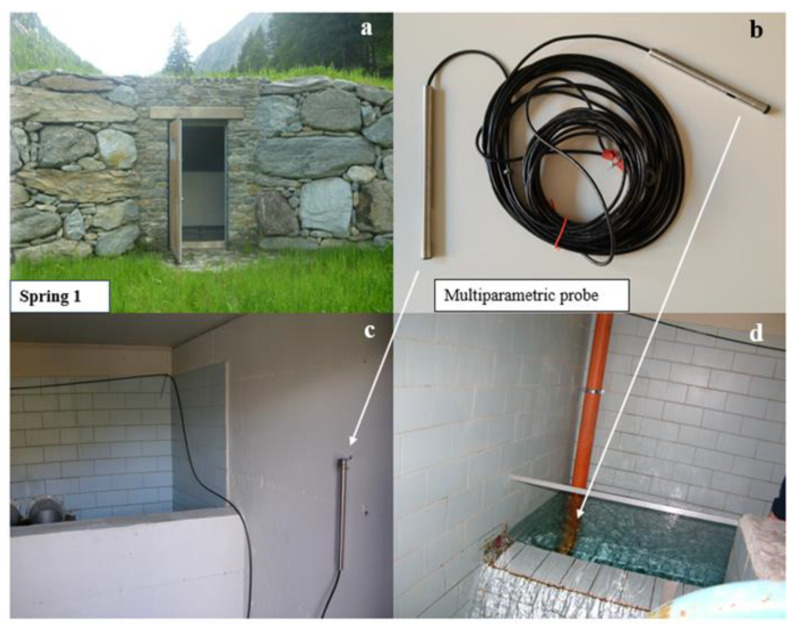
Mascognaz 1 spring’s structure and set up: (**a**) External view of the spring water intake structure; (**b**) Multiparametric probe geometry; (**c**) Installation of probe data logger; (**d**) Installation of recording probe**.**

Both Mascognaz 1 and 2 springs have been equipped with a water intake structure, which has the role of gathering surrounding groundwater, which nearly come to surface upstream of the spring infrastructure, using sub-horizontal drains, and conveying it inside the groundwater collection structure. Then, water flows through a rectangular weir into another pool, where water finally flows into the aqueduct pipe. Figure 3 represents the installation position of the OTT CTD multiparametric probe, which is composed of the datalogger and battery tube and the probe enclosing different sensors.

The OTT CTD groundwater datalogger is designed to record groundwater flow rate (Q) and temperature (T) values, as well as the electrical conductivity (EC) (Table 3). The probe, equipped with a water level transducer, transforms the hydrostatic pressure of the water column by means of an electrical signal that varies according to the deformation that the force in question produces on the sensor membrane component. Erroneous measurement results due to atmospheric air pressure fluctuations are eliminated. In order to translate the water level into discharge information, the formula for rectangular contracted weir, visible in Figure 3d, can be exploited according to the geometry of the spring gathering pool [38].

The OTT CTD measures the specific electrical conductivity using a 4-electrode conductivity sensor with an integrated temperature sensor. The measurement electrodes are made of graphite.

The described device setting allows recording of values hourly. As it has not been provided with a SIM for simultaneous records transmission, every year the research staff of Applied Geology in DIATI Department of Politecnico di Torino manually download data directly from the OTT probe on site.

## 3. Results

**Figure 4:**
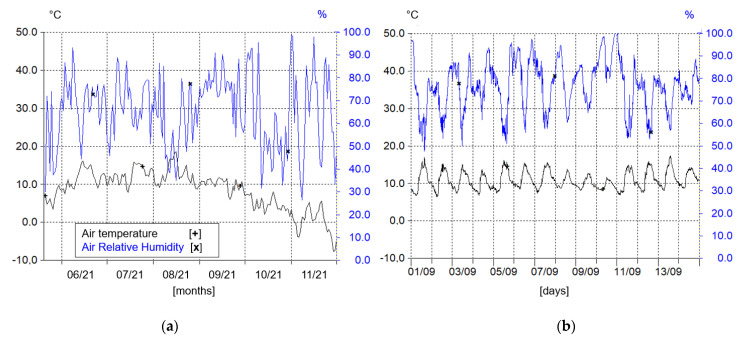

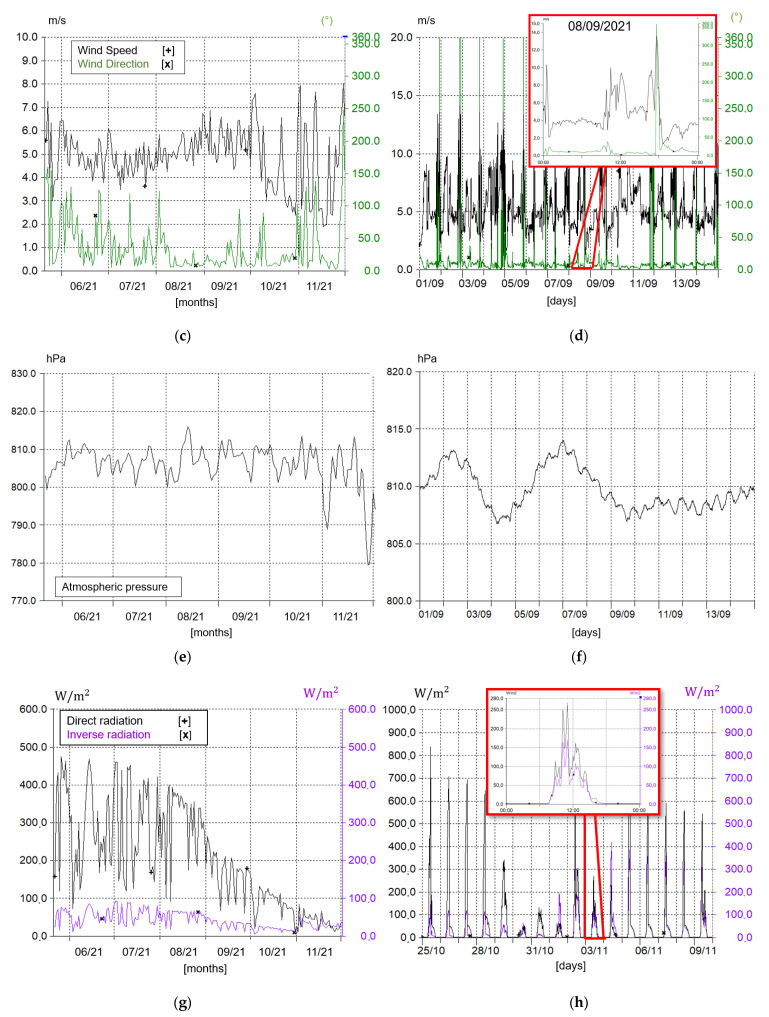

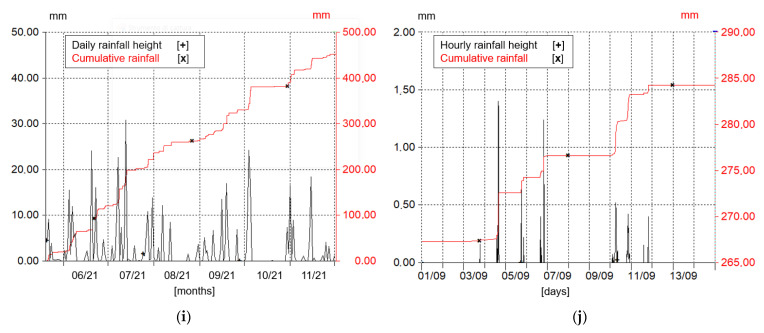
Graphs representing the parameters recorded from weather station sensors (Hydras 3 software layout): (**a**,**c**,**e**,**g**,**i**) Day mean values; (**b**,**d**,**f**,**h**,**j**) The real values registered with the time interval of 10 min. For precipitation results, it is plotted are the total daily rainfall height (**i**) and the total hourly rainfall height (**j**) with their cumulative.

Seven month time series of atmospheric and rainfall parameters (Figure 4a,c,e,g,i) were plotted to underline the potentiality of using the described instruments to analyze the Mascognaz basin weather conditions on a seasonal-temporal scale. Contrariwise, Figure 4b,d,f,h,j show the excepts of the same parameters with high time resolution (10 min time steps), demonstrating the equipment’s ability to analyze meteorological features in a short time, such as daily and hourly periods.

In detail, air temperature and air RH values, recorded by the hygrometer, follow the seasonality of the conditions recorded, showing a decrease in temperature below zero toward winter months (Figure 4a). The insight of the month of September, represented in Figure 4b, reflects the day/night oscillation of RH and temperature values better. CWS records the same two parameters with a very low discrepancy, since it is characterized by higher overall uncertainty for both RH and temperature with respect to the hygrometer sensor, it is mainly exploited for measurements cross-checking.

The CWS sonic anemometer results are shown in Figure 4c,d. The average daily wind direction on the left graph suggests the prevalence of wind blowing from the North/East side during the period, with average intensity ranging from 2 to 8 m/s. On the right side, a detailed plot for September shows some peaks in wind velocity up to 13 m/s with wind direction variating in all directions. It is worth noting that a 360 °C direction is equivalent to 0 °C, meaning North side wind direction.

Barometric pressure sensor (61302) results are represented in Figure 4e,f, as the aforementioned results. The left graph (Figure 4e) shows the daily average values on a bigger scale period, while in the right-side graph (Figure 4f) the direct value is captured with a time step of 10 min. For the same reason as RH and temperature, CWS atmospheric pressure sensor is applied for checking measurements of this parameter, increasing the quality of the analysis by giving more redundancy to the monitoring system. 

Albedometer records underline the direct and inverse radiation. The direct radiation value investigates the sky condition according to the season and cloud coverage. For instance, it turns out to be simple to recognize a decrease in radiation intensity during winter months (Figure 4g). The inverse radiation investigates the soil coverage that reflects the solar radiation and the reflection coefficient given by the wave angle impact. It is interesting to note that in the winter season, inverse radiation gets close to the direct one, due to the presence of powder snow that has a very high albedo value (0.8–0.9) [39] (Figure 4h).

The rainfall season pattern, detected by the weight pluviograph, represented in Figure 4i, is not showing the average daily value as the other parameters already described, but rather the total daily precipitation fell. Also, in Figure 4j, the total rainfall millimeter fallen within an hour time interval is presented. Rainfall plots are shown with a cumulative depiction.

**Figure 5:**
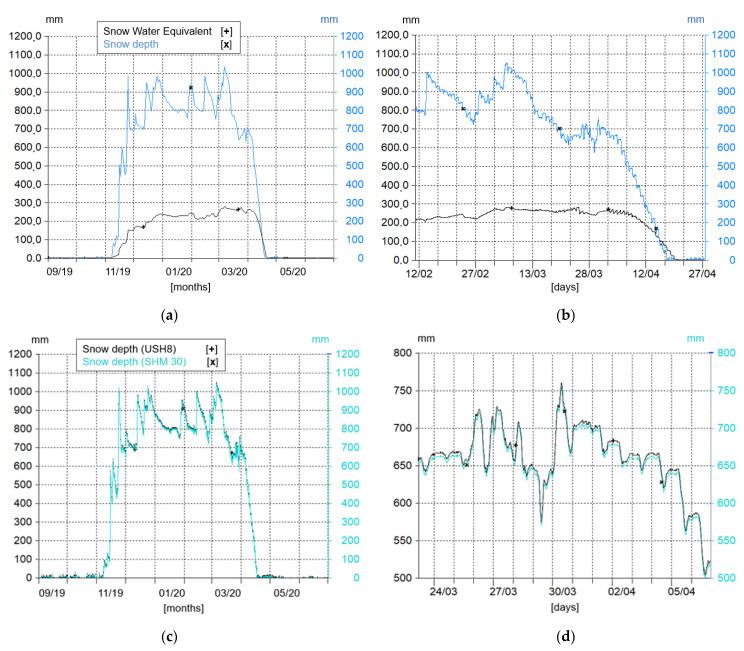
Graphs representing the parameters recorded from snowpack station sensors (Hydras 3 software layout): (**a**,**c**) Are day mean values; (**b**,**d**) Are the real values registered with the time interval of 10 min.

Figure 5a shows snow mantle characteristics from the snow scale and ultrasonic sensors during the winter season across 2019 and 2020. It is possible to notice that the depth of the snow layer is much higher than the snow water equivalent detected by the snow scale. That is due to the lower density of snow with respect to water. Snow density evolution can be analyzed through the ratio between these two parameters [40]: this information is essential to understanding the snowpack transformations across the season.

Figure 5b shows the real values detected by the two sensors along some days of spring, in which the snow mantle starts to melt until it completely disappears. Ultrasonic sensor (USH-8) and laser sensor (SHM 30) detect the same parameter (snow depth), and both are useful to verify the values recorded. Their comparison is represented in Figure 5c from a seasonal point of view and in Figure 5d on a more detailed scale. The latter one shows the same records with a slight difference in acquired values by the two sensors (around 5 mm). This systematic error could be erased by employing a further calibration of the two sensors by exploiting a third instrument record. Nevertheless, the overall uncertainty lower than 1 cm for snowpack, expresses a representative measurement for snow layer evolution along time.

**Figure 6:**
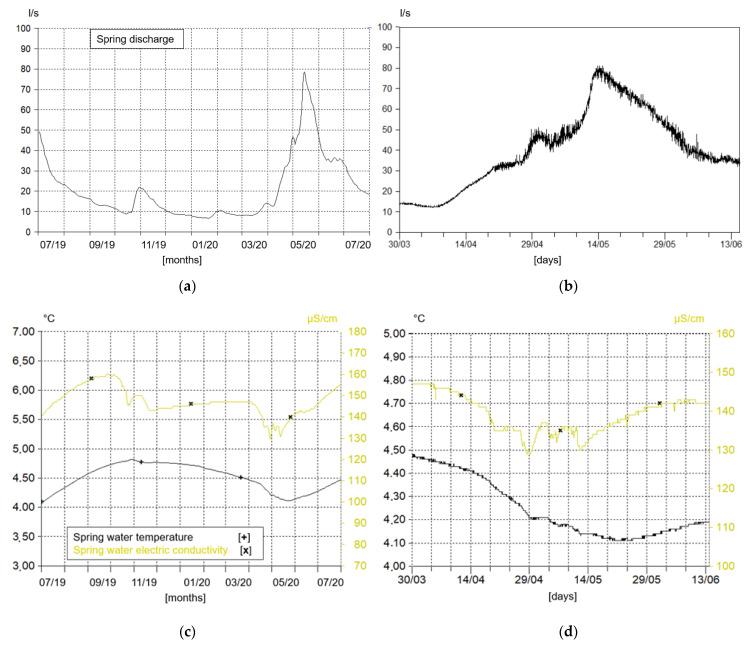
Graphs representing the parameters recorded from spring outflow sensor (Hydras 3 software layout): (**a**,**c**) Are day mean values; (**b**,**d**) Are the real values registered with the time interval of 1 h.

Figure 6a represents a one-year hydrograph read by the OTT multiparametric probe within the spring outflow reservoir. The recorded values have already been converted into the discharge data and averaged according to the daytime step. The direct value of discharge is recorded on a more detailed period as visualized in Figure 6b. It is detected every hour, instead of 10 min, as values are gathered by hydrometric and snowpack stations. Figure 6c,d represent the other two parameters recorded by OTT multiparametric probe, water temperature and water electric conductivity, which are strictly dependent on the features of the water circulation system. In fact, the lowering of both parameters during the snow melting season is visible (March–April) [41].

## 4. Discussion

In the previous section, to bring to light the potential of the SWS and hydrometric stations from the point of view of the information collected and processed, detected time series related to different variables were shown. The data collected allow us to describe not only the meteorological local-system conditions but also to guarantee the understanding of the hydrogeological system of the considered mountain basin, by considering the interaction between precipitations and snowpack melting with the spring hydrometric behaviors.

**Figure 7:**
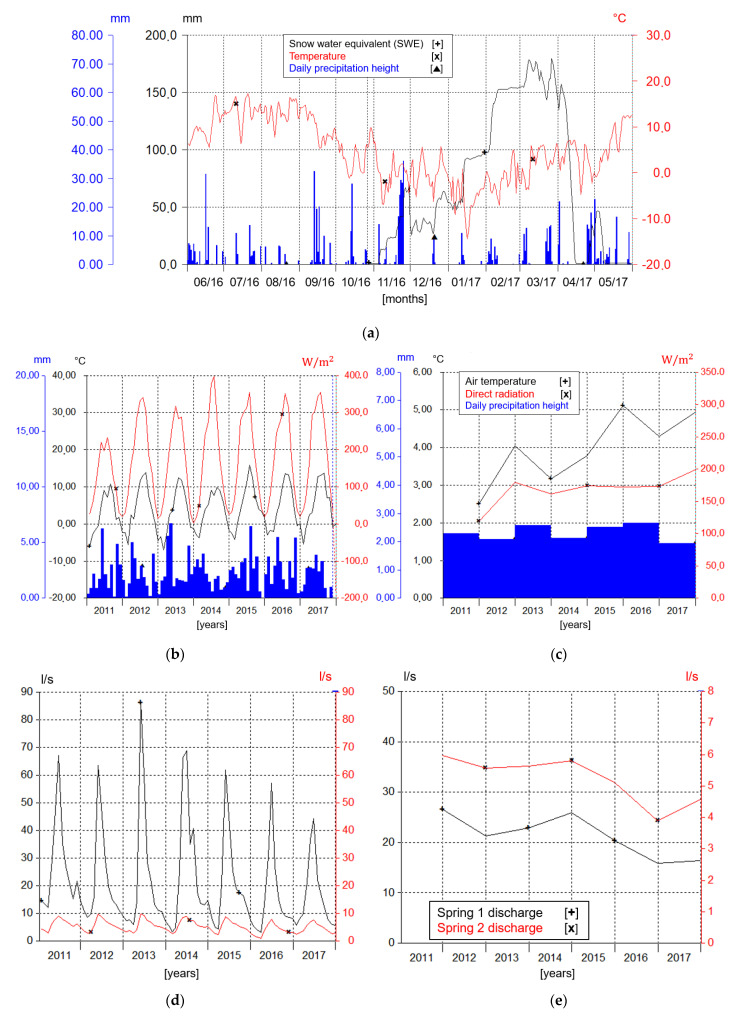
(**a**) Daily means time series of SWE, temperature, and daily precipitations. (**b**,**c**) Monthly means and yearly mean values of air temperature, direct radiation, and daily precipitations height. (**d**,**e**) Monthly means and yearly means values of discharge were detected in both springs. (Hydras 3 software layout).

As reported in Figure 7a, the intersection between daily precipitations, temperature and SWE graphs allow an understanding of the quality of such precipitations (liquid-solid) and how much they increase the thickness of the snowpack. Furthermore, the development of the snow layer, affected by multiple factors including temperature and precipitation, can be easily observed from the same representation. During spring, it is visible from the sensors recording an increase in temperatures, which are responsible for the total melting of the snow mantle.

As already observed in Figure 5a, the evolution of the snowpack during the autumn-winter-spring seasons can be correctly investigated using the parameters of SWE (Snow Scale) and snow depth (USH8, SHM 30), since their ratio multiplied by the specific weight of the water (1000 kg/m^3^) gives back the density of the snow, the best indicator to understand the average metamorphism state of the snow layer. The increase in density occurs up to the moment in which the snow coincides almost with the ice state so that a further phase of wet metamorphism leads to melting and dispersion of water from the snowpack. Moreover, different constructive, destructive, and wet metamorphism behaviors of snow are visible when the decrease in snow depth occurs, even though it is not accompanied by a simultaneous decrease in SWE.

In addition to the aforementioned considerations, using the Mascognaz station instruments and sensors set-up, it is possible to observe the variation of several parameters with a multi-year time series (Figure 7b,c), where the annual and monthly averages starting from 2011 until 2018 are plotted. The annual means show a slight increase in temperatures (hygrometer) accompanied by a slight increase in the intensity of solar radiation (albedometer), reflecting characteristics attributable to global warming [42]. The depicted evolution of climatic conditions can be coupled with the effects observed by the hydrogeological system, described by the spring flow data (Figure 6) The system has illustrated the variation in atmospheric, pluviometric and snowpack conditions by showing a progressively reduction of the last one. Although the series of average daily precipitations (Figure 7b,c) was found to be rather flat in the same years, a smaller number of rain events with greater intensity were estimated from the analysis of the data. This can lead to understanding more precisely the intrinsic characteristics of the aquifer system and how it relates to the rainfall regime. Therefore, according to the necessity of territorial planning improvement for water resource knowledge and management, this case can provide very useful information in anticipation of further changes in weather conditions. 

Another important element provided by multiparametric probes installed to Mascognaz 1 and 2 springs is the spring hydrograph over several years, displaying the spring outflow rate data using the monthly averages (Figure 7d). This can allow for the analysis of different hydrogeological system features, such as the aquifer structure, recharge and discharge phases, the recession period, hydric input dependency, and water circulation assessment [43].

**Figure 8:**
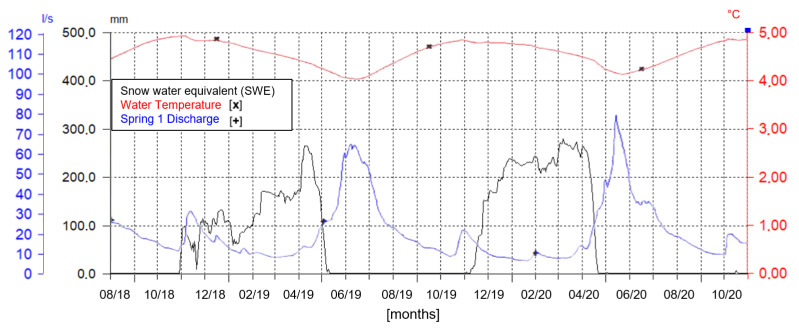
Daily means time series of SWE, water temperature and Spring 1 discharge rate from summer 2018 to autumn 2020 (Hydras 3 software layout).

The double year plot (Figure 8) shows that the discharge rate is highly dependent on snow melting water input during the spring season, while in autumn the hydrograph shows a slight increase due to summer rainfalls. In fact, with the decreasing snowpack depth along seasons, the reduction of aquifer discharge is clearly visible (Figure 7d,e). Therefore, this makes the water resource coming from melting activity the most important component, according to the recharge effectiveness of the aquifer. To remark this phenomenon, the water temperature parameter plotted in the same graph (Figure 8) shows its oscillation from warmer values after summer to colder ones during spring due to the snow melting activity.

This fact states the importance of snowpack sensors, which permit observation of time lag intercurrent between mantle disappearance and spring recharge phase. Moreover, they reveal the correspondence between maximum snow depth values and water discharge peak values.

The density of data acquired allows the visualization of hourly and daily oscillation, while the long series leads to the observation of how the water resource changes over time. In fact, the statistical significance of variable trends assessment increase as much as the series is dense. For each sensor of the station, a recording frequency has been chosen that allows elaboration of around 88,000 values every 5 years, to perform reliable statistical tests, even though the series has a length of a few years.

## 5. Conclusions

The impacts on hydrogeological recharge mechanisms in alpine climate systems deriving from climate change are still largely unknown. Examining variations in groundwater mechanisms in response to both climate-driven and anthropogenic effects is increasingly crucial for realizing correct estimates of water availability even in the longer term.

Data from the combination of weather station sensors with spring flow-rate instruments, installed in the alpine Mascognaz basin, can guarantee an entire understanding of how one set of parameters can affect other results, defining consequential cause-and-effect relationships.

In this case study, the importance of the snow melting component for the porous aquifer recharge is shown. The slow and constant rate of water release from the mantle increases the infiltration effectiveness. 

Temperature and solar radiation increasing prospectives announce that the snow mantle will decrease in the next decade, directly causing a decrease in aquifer water storage. This can be helpful for mountain authorities in order to understand which factors most affect groundwater dynamics in a particular area and to plan actions for its better management. In this case, the acknowledgement of future exploitation of aquifers less affected by snow melting recharge should be beneficial in terms of the decision-making process. 

Both graphical and statistical data interpretation can be crucial for governance actions to respond to environmental variations driven by climate change. In fact, since a large part of Alpine groundwater bodies are exploited with the role of drinking water, it is important to define strategies for understanding their evolution over time, making the right economic investments aimed at using them according to forecasted predictions and sustainable development goals.

## Figures and Tables

**Table 1:** Weather station sensors description.

Parameter	Sensor	Characteristics
Air temperature (°C)	hygrometer HygroClip2 (HC2-S3) (Rotronic AG, Bassersdorf, Switzerland)	range of application: −50 to 100 °C operating temperature: −50 to 100 °C housing:polycarbonate overall uncertainty: +/−0.1 °C
Air temperature (°C)	CWS All-In-One (AIO) Weather Sensor (Climatronics Corporation, New York, NY, USA)	range of application: −40 to 50°C operating temperature: −50 to 70°C overall uncertainty: +/−0.2°C
Relative humidity (%)	hygrometer HygroClip2 (HC2-S3) (Rotronic AG, Bassersdorf, Switzerland)	range of application: 0–100% RH operating temperature: −50 to 100 °C housing:polycarbonate overall uncertainty: +/−0.8% on measured value
Relative humidity (%)	CWS All-In-One (AIO) Weather Sensor (Climatronics Corporation, New York, NY, USA)	range of application: 0–100% RH operating temperature: −50 to 70°C overall uncertainty: +/−3% on measured value
Wind speed (m/s)	CWS All-In-One (AIO) Weather Sensor (Climatronics Corporation, New York, NY, USA)	range of application: 0–50 m/s operatingtemperature: −50 to 70 °C overall uncertainty: +/−0.1 m/s
Wind direction (°)	CWS All-In-One (AIO) Weather Sensor (Climatronics Corporation, New York, NY, USA)	range of application: 0 to 360° operating temperature: −50 to 70 °C overall uncertainty: +/−2°
Direct and inverse radiation (W/m²)	Albedometer model CMA6 (Kipp&Zonen, Delft, The Netherlands)	range of application: 0–2000 W/m² operating temperature: −40 to 80 °C overall uncertainty: +/−20 W/m²
Atmospheric pressure (hPa)	Barometric pressure sensor model 61302 (Young Co, Traverse City, MI, USA)	range of application: 500 to 1100 hPa operating temperature: −50 to 60 °C overall uncertainty: +/−0.2 hPa
Atmospheric pressure (hPa)	CWS All-In-One (AIO) Weather Sensor (Climatronics Corporation, New York, NY, USA)	range of application: 600 to 1100 hPa operating temperature: −50 to 70 °C overall uncertainty: +/−0.35 hPa
Rainfall (cumulate, mm; intensity, mm/h)	Pluviograph model PLUVIO2 OTT Hydromet, Kempten, Germany)	gathering area: 200 cm^2^ gathering volume: 1500 mm operating temperature: −40 to 60 °C resolution: +/−0.01 mm overall uncertainty: +/−0.1 mm
Rainfall/snow melting (mm)	Underground pluviograph model DQA030 (LSI Lastem, Settala, Italy)	gathering area: 324 cm^2^ operating temperature: 0 to 50 °C housing: Inox AISI304 resolution:+/−0.2 mm overall uncertainty: 0 to +/−1 mm/min

**Table 2:** Sommer GmbH & Co. KG snowpack station sensors.

Parameter	Sensor	Characteristics
Snow water equivalent (mm)	Snow scale Snow pillow (Sommer GmbH & Co KG, Koblach, Austria)	range of application: 0 to 2500 mm operating temperature: > −30 °C material: fabric thickness PVC-foil resolution: 1 mm overall uncertainty: 0.25% +/− on measured value
Snow depth (mm)	Ultrasonic sensor USH-8 (Sommer GmbH & Co KG, Koblach, Austria)	range of application: 0 to 8000 mm operating temperature: −35 to 60 °C housing: aluminum resolution: +/−1 mm overall uncertainty: +/−0.10% on measured value
Snow depth (mm)	Laser sensor model SHM 30 (G. Lufft Mess-und Regeltechnik GmbH, Fellbach, Germany)	range of application: 0 to 10 m distance range: 0.1 to 30 m operating temperature: −40 to 50 °C resolution: +/−0.1 mm overall uncertainty: +/−5 mm

**Table 3:** Spring outflow sensors.

Parameter	Sensor	Characteristics
Water level (mm)	Multiparametric probe OTT CTD (OTT HydroMet GmbH, Kempten, Germany)	range of application: 0 to 4 m operating temperature: −40 °C to 85 °C resolution: +/−0.1 cm overall uncertainty: +/−0.05% FS
Water temperature (°C)	Multiparametric probe OTT CTD (OTT HydroMet GmbH, Kempten, Germany)	range of application: −25 to 70 °C operating temperature: −40 °C to 85 °C resolution: +/−0.01 °C overall uncertainty: +/−0.1 °C
Water electric conductivity (μS/cm)	Multiparametric OTT CTD (OTT HydroMet GmbH, Kempten, Germany)	range of application: 1 to 2000 μS/cm operating temperature: −40 °C to 85 °C resolution: +/−1 μS/cm overall uncertainty: 0.5 % on measured value

## Data Availability

The hourly recorded data used to support the findings of this study have not been made directly available because they are the property of Politecnico di Torino. However, they are reported as graphs throughout this study.

## References

[1] IGRAC (International Groundwater Resources Assessment Centre) (2021). Transboundary Aquifers of the World [Map], 2021 ed., Scale 1:50,000,000.

[2] Boretti A., Rosa L. (2019). Reassessing the projections of the world water development report. NPJ Clean Water.

[3] Unesco (2019). Leaving No One Behind, The United Nations World Water Development Report 2019.

[4] Lo Russo S., Amanzio G., Ghione R., De Maio M. (2015). Recession Hydrographs and Time Series Analysis of Springs Monitoring Data: Application on Porous and Shallow Aquifers in Mountain Areas (Aosta Valley). Environ. Earth Sci..

[5] Amanzio G., Marchionatti F., Lavy M., Ghione R., De Maio M. (2016). Springs monitoring data analysis with a frequency and time domain approach: The case study of Mascognaz spring (Aosta Valley). Geoing. Ambient. E Min..

[6] Lo Russo S., Suozzi E., Gizzi M., Taddia G. (2021). SOURCE: A Semi-Automatic Tool for Spring-Monitoring Data Analysis and Aquifer Characterisation. Environ. Earth Sci..

[7] Wu W.Y., Lo M.H., Wada Y., Famiglietti J.S., Reager J.T., Yeh P.J.F., Ducharne A., Yang Z.L. (2020). Divergent Effects of Climate Change on Future Groundwater Availability in Key Mid-Latitude Aquifers. Nat. Commun..

[8] Gizzi M., Mondani M., Taddia G., Suozzi E., Lo Russo S. (2022). Aosta Valley Mountain Springs: A Preliminary Analysis for Understanding Variations in Water Resource Availability under Climate Change. Water.

[9] Carvalho-Santos C., Monteiro A.T., Azevedo J.C., Honrado J.P., Nunes J.P. (2017). Climate Change Impacts on Water Resources and Reservoir Management: Uncertainty and Adaptation for a Mountain Catchment in Northeast Portugal. Water Resour. Manag..

[10] Amanambu A.C., Obarein O.A., Mossa J., Li L., Ayeni S.S., Balogun O., Oyebamiji A., Ochege F.U. (2020). Groundwater System and Climate Change: Present Status and Future Considerations. J. Hydrol..

[11] Berg N., Hall A. (2015). Increased Interannual Precipitation Extremes over California under Climate Change. J. Clim..

[12] Mo C., Ruan Y., He J., Jin J.L., Liu P., Sun G. (2019). Frequency Analysis of Precipitation Extremes under Climate Change. Int. J. Climatol..

[13] Szwed M. (2019). Variability of Precipitation in Poland under Climate Change. Theor. Appl. Climatol..

[14] Barnett T.P., Adam J.C., Lettenmaier D.P. (2005). Potential Impacts of a Warming Climate on Water Availability in Snow-Dominated Regions. Nature.

[15] Clow D.W. (2010). Changes in the Timing of Snowmelt and Streamflow in Colorado: A Response to Recent Warming. J. Clim..

[16] Jepsen S.M., Molotch N.P., Williams M.W., Rittger K.E., Sickman J.O. (2012). Interannual Variability of Snowmelt in the Sierra Nevada and Rocky Mountains, United States: Examples from Two Alpine Watersheds. Water Resour. Res..

[17] Wu X., Che T., Li X., Wang N., Yang X. (2018). Slower Snowmelt in Spring Along With Climate Warming Across the Northern Hemisphere. Geophys. Res. Lett..

[18] Jódar J., González-Ramón A., Martos-Rosillo S., Heredia J., Herrera C., Urrutia J., Caballero Y., Zabaleta A., Antigüedad I., Custodio E. (2020). Snowmelt as a Determinant Factor in the Hydrogeological Behaviour of High Mountain Karst Aquifers: The Garcés Karst System, Central Pyrenees (Spain). Sci. Total Environ..

[19] Thibert E., Dkengne Sielenou P., Vionnet V., Eckert N., Vincent C. (2018). Causes of Glacier Melt Extremes in the Alps Since 1949. Geophys. Res. Lett..

[20] Colucci R.R., Guglielmin M. (2019). Climate Change and Rapid Ice Melt: Suggestions from Abrupt Permafrost Degradation and Ice Melting in an Alpine Ice Cave. Prog. Phys. Geogr..

[21] Mohammadi Z., Shoja A. (2014). Effect of Annual Rainfall Amount on Characteristics of Karst Spring Hydrograph. Carbonates Evaporites.

[22] Kodali R.K., Mandal S. IoT Based Weather Station. Proceedings of the 2016 International Conference on Control, Instrumentation, Communication and Computational Technologies (ICCICCT).

[23] Dombrowski O., Franssen H.H., Brogi C. (2021). Performance of the ATMOS41 All-in-One Weather Station for Weather Monitoring. Sensors.

[24] Attah D.A., Bankkole G.M. (2012). Time Series Analysis Model for Annual Rainfall Data in Lower Kaduna Catchment Kaduna, Nigeria. Glob. J. Res. Eng..

[25] Olatayo T.O., Taiwo A.I. (2014). Statistical Modelling and Prediction of Rainfall Time Series Data. Glob. J. Comuter Sci. Technol..

[26] Zhang Y., Yu Y., Peng M., Meng R., Hu K., Yu C. (2018). Temporal and Seasonal Variations of Mortality Burden Associated with Hourly Temperature Variability: A Nationwide Investigation in England and Wales. Environ. Int..

[27] Harris I., Osborn T.J., Jones P., Lister D. (2020). Version 4 of the CRU TS Monthly High-Resolution Gridded Multivariate Climate Dataset. Sci. Data.

[28] Cerino Abdin E., Taddia G., Gizzi M., Lo Russo S. (2021). Reliability of Spring Recession Curve Analysis as a Function of the Temporal Resolution of the Monitoring Dataset. Environ. Earth Sci..

[29] Lucianetti G., Penna D., Mastrorillo L., Mazza R. (2020). The role of snowmelt on the spatio-temporal variability of spring recharge in a dolomitic mountain group, Italian Alps. Water.

[30] Brkić Ž., Kuhta M., Hunjak T. (2018). Groundwater flow mechanism in the well-developed karst aquifer system in the western Croatia: Insights from spring discharge and water isotopes. Catena.

[31] Diodato N., Guerriero L., Fiorillo F., Esposito L., Revellino P., Grelle G., Guadagno F.M. (2014). Predicting monthly spring discharges using a simple statistical model. Water Resour. Manag..

[32] Negi G., Joshi V. (2004). Rainfall and spring discharge patterns in two small drainage catchments in the Western Himalayan Mountains, India. Environmentalist.

[33] Jan F., Min-Allah N., Saeed S., Iqbal S.Z., Ahmed R. (2022). IoT-Based Solutions to Monitor Water Level, Leakage, and Control for Smart Water Tanks. Water.

[34] Jan F., Min-Allah N., Düştegör D. (2021). IoT based smart water quality monitoring: Recent techniques, trends and challenges for domestic applications. Water.

[35] Crepaldi S., De Maio M., Suozzi E. (2015). Preliminary Study on the Snow-Melt for the Groundwater Recharge Estimated by an Advanced Meteorological Station. Eng. Geol. Soc. Territ..

[36] Varhola A., Wawerla J., Weiler M., Coops N.C., Bewley D., Alila Y. (2010). A New Low-Cost, Stand-Alone Sensor System for Snow Monitoring. J. Atmos. Ocean. Technol..

[37] Fiorillo F., Doglioni A. (2010). The Relation between Karst Spring Discharge and Rainfall by Cross-Correlation Analysis (Campania, Southern Italy). Hydrogeol. J..

[38] Swamee P.K. (1988). Generalized rectangular weir equations. J. Hydraul. Eng..

[39] Dirmhirn I., Eaton F.D. (1975). Some Characteristics of the Albedo of Snow. J. Appl. Meteorol. Climatol..

[40] López-Moreno J.I., Fassnacht S.R., Heath J.T., Musselman K.N., Revuelto J., Latron J., Morán-Tejeda E., Jonas T. (2013). Small Scale Spatial Variability of Snow Density and Depth over Complex Alpine Terrain: Implications for Estimating Snow Water Equivalent. Adv. Water Resour..

[41] Zuecco G., Carturan L., De Blasi F., Seppi R., Zanoner T., Penna D., Borga M., Carton A., Dalla Fontana G. (2019). Understanding Hydrological Processes in Glacierized Catchments: Evidence and Implications of Highly Variable Isotopic and Electrical Conductivity Data. Hydrol. Process..

[42] Pandey C.K., Katiyar A.K. (2013). Solar Radiation: Models and Measurement Techniques. J. Energy.

[43] Kresic N., Bonacci O. (2010). Spring Discharge Hydrograph. Groundwater Hydrology of Springs.

